# Proteomics analysis and proteogenomic characterization of different physiopathological human lenses

**DOI:** 10.1186/s12886-017-0642-9

**Published:** 2017-12-19

**Authors:** Xiaohang Wu, Zhenzhen Liu, Xiayin Zhang, Dongni Wang, Erping Long, Jinghui Wang, Wangting Li, Weiyi Lai, Qianzhong Cao, Kunhua Hu, Weirong Chen, Haotian Lin, Yizhi Liu

**Affiliations:** 1 State Key Laboratory of Ophthalmology, Zhongshan Ophthalmic Center, Sun Yat-sen University, 54# Xianlie Road, Guangzhou, Guangdong 510060 China; 2 Department of Pharmacology, Zhongshan School of Medicine, Sun Yat-sen University, Guangzhou, Guangdong 510080 China

**Keywords:** Human lens, Regenerative lenses with secondary cataract (RLSC), Proteomics, Proteogenomic analysis

## Abstract

**Background:**

The aim of the present study was to identify the proteomic differences among human lenses in different physiopathological states and to screen for susceptibility genes/proteins via proteogenomic characterization.

**Methods:**

The total proteomes identified across the regenerative lens with secondary cataract (RLSC), congenital cataract (CC) and age-related cataract (ARC) groups were compared to those of normal lenses using isobaric tagging for relative and absolute protein quantification (iTRAQ). The up-regulated proteins between the groups were subjected to biological analysis. Whole exome sequencing (WES) was performed to detect genetic variations.

**Results:**

The most complete human lens proteome to date, which consisted of 1251 proteins, including 55.2% previously unreported proteins, was identified across the experimental groups. Bioinformatics functional annotation revealed the common involvement of cellular metabolic processes, immune responses and protein folding disturbances among the groups. RLSC-over-expressed proteins were characteristically enriched in the intracellular immunological signal transduction pathways. The CC groups featured biological processes relating to gene expression and vascular endothelial growth factor (VEGF) signaling transduction, whereas the molecular functions corresponding to external stress were specific to the ARC groups. Combined with WES, the proteogenomic characterization narrowed the list to 16 candidate causal molecules.

**Conclusions:**

These findings revealed common final pathways with diverse upstream regulation of cataractogenesis in different physiopathological states. This proteogenomic characterization shows translational potential for detecting susceptibility genes/proteins in precision medicine.

**Electronic supplementary material:**

The online version of this article (10.1186/s12886-017-0642-9) contains supplementary material, which is available to authorized users.

## Background

The eye lens has the most abundant protein content within the body, with proteins accounting for more than 35% of its wet weight [1]. The disruption of the normal quality or quantity of lens constitutional proteins leads to the opacification of the refractive structure, namely, cataracts, which are the leading cause of blindness worldwide [2]. Cataracts can be induced under a variety of physiopathological states, including (but not limited to) age-related cataracts (ARCs) and congenital cataracts (CCs). Although different types of cataracts share common final alterations of lens crystallin proteins in terms of their quality (structure) and quantity [3, 4], the complicated predisposing factors (including genetics and environmental stimuli) and the upstream regulatory mechanisms are highly diverse and have not been thoroughly studied. Genetic alterations account for less than 30% percent of cataractogenesis events in CCs, and far fewer genetic associations have been detected in ARCs [5].

In a recent study, we achieved functional lens regeneration in CC patients after a novel minimally invasive surgical procedure [6]. The included subjects were tested in advance to exclude cataractogenetic genomic variants. Unfortunately, three cases still exhibited evident secondary opacification of the regenerative lenses more than 2 years after primary surgery. The unique regenerative lens with secondary cataract (RLSC) model has enable the identification of a novel proteome composition and the characterization of a novel molecular mechanism underlying the predisposition to cataractogenesis [7, 8].

An overwhelming majority of previous cataract proteomics studies have exclusively focused on crystallin proteins; thus, little is known about non-crystallin proteins, which are expressed at significantly lower levels but still play key roles in the maintenance of lens function [9]. Isobaric tagging for relative and absolute protein quantification (iTRAQ) is a recently developed technology for quantitative proteomics that enables for high-throughput identification and relative quantification of trace amounts of protein [10]. Moreover, the widely performed whole exome sequencing (WES) methodology can identify numerous genetic variants as potential origins of protein alterations [11, 12]. By combining these techniques, this proteogenomic characterization is expected to narrow the list of candidate causal molecules. Therefore, we conducted a preliminary proteomics comparative analysis of RLSC, CC and ARC with respect to different physiopathological states, using iTRAQ, followed by WES of representative individuals. These findings will update our current knowledge of the molecular mechanism responsible for cataractogenesis and lens development. Furthermore, predictive therapeutic-directed pathways or molecules may be discerned by combining the genetic and proteomics perspectives.

## Methods

### Sample collection

The regenerative, CC and ARC lenses were obtained from the Zhongshan Ophthalmic Center (ZOC) in Guangzhou, China. As a group of control, normal human lenses were obtained from the Eye Bank of Guangdong Province. The study protocol conformed to the ethical guidelines of the 1975 Declaration of Helsinki and was approved by the Ethical Review Committee of ZOC. Written informed consent was obtained from all subjects (or from at least one guardian of each participating child).

CC and ARC subjects were examined and confirmed by at least three experienced ophthalmologists according to the International Classification of Diseases and Codes. The lens contents were collected during cataract extraction.

The regenerative lenses were obtained using novel minimally invasive surgical methods of cataract removal developed by our group, which preserved the endogenous lens epithelial stem cells (LECs) and produced regenerative lenses in situ with improved visual function. Unfortunately, three cases exhibited evident secondary total opacification of the regenerative lenses beyond 2 years after primary surgery. Thus, secondary cataract extraction surgery was performed as a routine approach (irrigation and lens aspiration [I/A] with no phacoemulsification energy administered) with intraocular lens implantation, during which the regenerated lens contents were collected. The baseline information of the included subjects is presented in Table 1. All samples were immediately stored at −80 °C until further analysis.

**Table 1:** Baseline information of the included subjects

Group	Sample No.	Age (years)	Gender	Cataract morphology	iTRAQ labelling	WES
RLSC	1	2	Male	NA	113	Yes
	2	2	Male	NA	114	Yes
	3	2	Female	NA	115	No
CC	4	2	Male	Nuclear	116	Yes
	5	2	Male	Nuclear	117	No
	6	3	Female	Nuclear	118	No
ARC	7	63	Female	Nuclear + cortical (LOCS II)	119	No
Normal lens	8	21	Male	NA	121	No

*RLSC* regenerative lenses with secondary cataract, *CC* congenital cataract, *ARC* aged-related cataract, *WES* whole exome sequencing

### Sample preparation and iTRAQ labelling (Fig. 1)

**Fig. 1:**
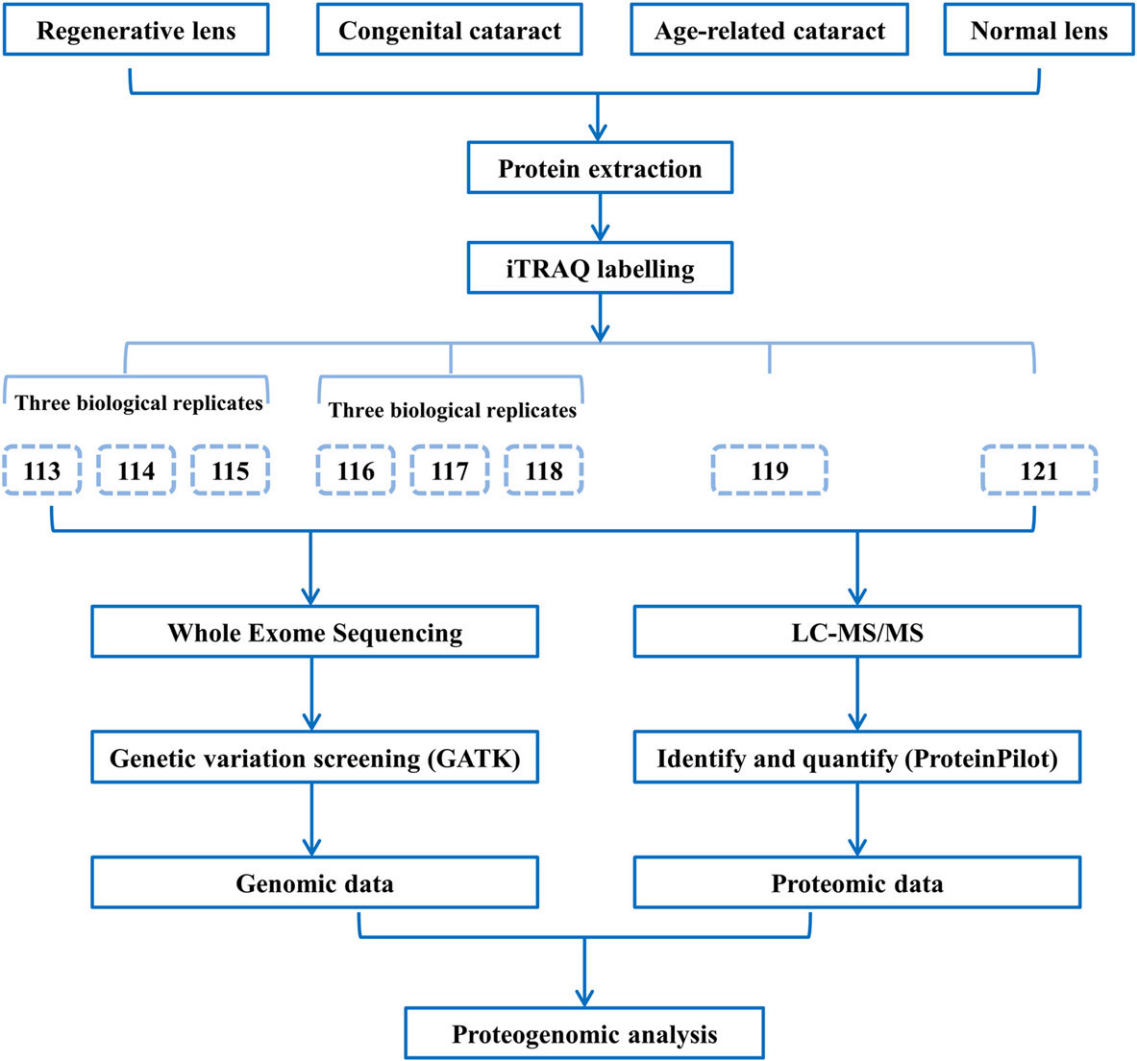
Flowchart of the proteogenomic analysis of the human RLSC, CC and ARC lenses

Eight lens samples from four groups were processed and individually examined. Protease inhibitor (Roche Complete ULTRA tablets, mini EASY pack) was added to each sample prior to the experiments. Cold acetone was subsequently added at 4 times the volume of the pellet, and the sample was precipitated at −20 °C overnight. The sample was subsequently centrifuged twice at 14,000 x g for 10 min at 4 °C. Subsequently, the pellet was air-dried, and protein lysis was performed in lysis buffer. The proteins were quantified using the GE 2-D Quant Kit according to the manufacturer’s instructions. Each sample containing 100 μg of quantified protein was enzymolyzed with trypsin and labelled with iTRAQ reagent using the iTRAQ Reagent-8Plex Multiplex Kit (AB SCIEX 4381663) according to the manufacturer’s instructions. The three regenerative lenses were labelled with iTRAQ reagents 113, 114, and 115; the three CC lenses were labelled with iTRAQ reagents 116, 117, and 118; and the ARC and normal lenses were labelled with iTRAQ reagents 119 and 121, respectively. Figure 1 illustrates the workflow of the detailed experiment procedures.

### Liquid chromatography tandem mass spectrometry (LC-MS/MS) and data analysis

The protein samples were separately measured using the Dionex Ultimate 3000 RSLCnano system. The pre-column was washed with the 0.1% formic acid and 2% acetonitrile. In each measurement, the peptides from a standard sample were loaded offline onto a 75-μm i.d. × 150 mm Acclaim PepMap RSLC C18 column (2 μm, 100 Å, nanoViper). The flow was diverted to the separation column at a flow rate of 300 nL/min. The peptides were separated and eluted with an effective gradient of 4-50% solution B (80% acetonitrile [ACN] and 0.1% formic acid) in a 65-min survey full scan, and the five most intense ions were selected for a zoom scan to determine the charge state using the Thermo Scientific Q Exactive Hybrid Quadrupole-Orbitrap™ Mass Spectrometer. Subsequently, MS/MS with higher-energy collisional dissociation (HCD) scans were acquired in an Orbitrap at a resolution of 17,500; the maximum ion accumulation time and target value were maintained at 60 ms and 4e^5^, respectively, and the normalized collision energy (NCE)**/** stepped NCE was 30.

ProteinPilot™ 5.0 software (AB SCIEX, Framingham, MA, USA, 2012), Paragon™ Algorithm 5.0.0.0, 4767 was subsequently used for protein identification and iTRAQ quantification. The database searching parameters were set as follows: trypsin as the digestion enzyme; iTRAQ 8plex as the sample type; no special factors; biological modification as ID focus; thorough as search effort; HUMAN_uniprot_2015.8.3.fasta as the database; false discovery rate analysis was conducted, and the detected protein threshold was set at less than 0.01; the detected protein threshold [Unused ProtScore (Conf)] cutoff was 1.3 (unused ProtScore), with at least two peptides with 95% confidence.

The identified proteins were statistically analyzed to filter for the deregulated proteins of each group compared to normal lens. The filtering steps include: 1) the difference between groups were compared using t-test and *p* < 0.05 was considered statistically significant; 2) the coefficient of variation (C.V) was used to evaluate the dispersion of the replicates within groups, and detected proteins with C.V **≤** 0.5 were considered reliable; 3) proteins with ratio of >20 or **<**0.5 were considered generated with unreliable signals and excluded; 4) protein ratios of ≥2 and **≤**0.5 represented significant up-regulation and down-regulation between the groups.

### Biological interpretation of the protein expression data

The significantly up-regulated proteins in each group were annotated according to the Gene Ontology (GO) database (https://david-d.ncifcrf.gov/summary.jsp) [13]. Pathway annotation of these proteins was conducted by searching against the Kyoto Encyclopedia of Genes and Genomes (KEGG) database (http://www.genome.jp/kegg/pathway.html). The significance of the association between the dataset and canonical pathways was measured as the ratio of the protein number from the dataset that maps to the canonical pathway and the *p*-value of the estimate. A functional regulatory network analysis of the common evaluated proteins from the three groups was performed using the web-based Search Tool for the Retrieval of Interacting Genes/Proteins (STRING) database (version 10.0, http://string.embl.de/). STRING was used to build the diagrams from numerous sources, including known experimental data and/or computational data based on genomic context analysis. The matching interactions were provided with a confidence score based on the reliability of the interaction.

### WES and data analysis

The genomic DNA from the blood samples of two subjects in the RLSC group and one subject in the CC group was extracted using Qiagen blood midi or maxi kits (Qiagen, Hilden, Germany) according to the manufacturer’s instructions. WES was performed at iGenostics Biotechnology (Guangzhou, China). All samples were processed using the Illumina HiSeq 2000 platform.

The human reference genome was GRCh37/hg19, and the RefGene database was downloaded from the University of California, Santa Cruz (UCSC). Read mapping and polymerase chain reaction (PCR) duplicate removal were performed using the Burrows-Wheeler Aligner (BWA), Sequence Alignment Map (SAM) Tools and Picard tools. The Genome Analysis Toolkit (GATK) was used for local realignment, base quality score recalibration and variant calling (HaplotypeCaller) with default parameters. Single-nucleotide polymorphism (SNP) and insertion or deletion (INDEL) variants were annotated using ANNOVAR. The candidate variations were manually reviewed using the Integrative Genomics Viewer (IGV).

## Results

### The most complete proteome identification of human lenses and comprehensive quantification of the distinguished expressed proteins in different physiopathological states

In total, 1251 proteins were identified across all lens samples, based on the detected protein threshold set at *p* < 0.05. A complete list of proteins identified with detailed information regarding their identification and quantification is provided in Additional file 1: Table S1.

**Fig. 2:**
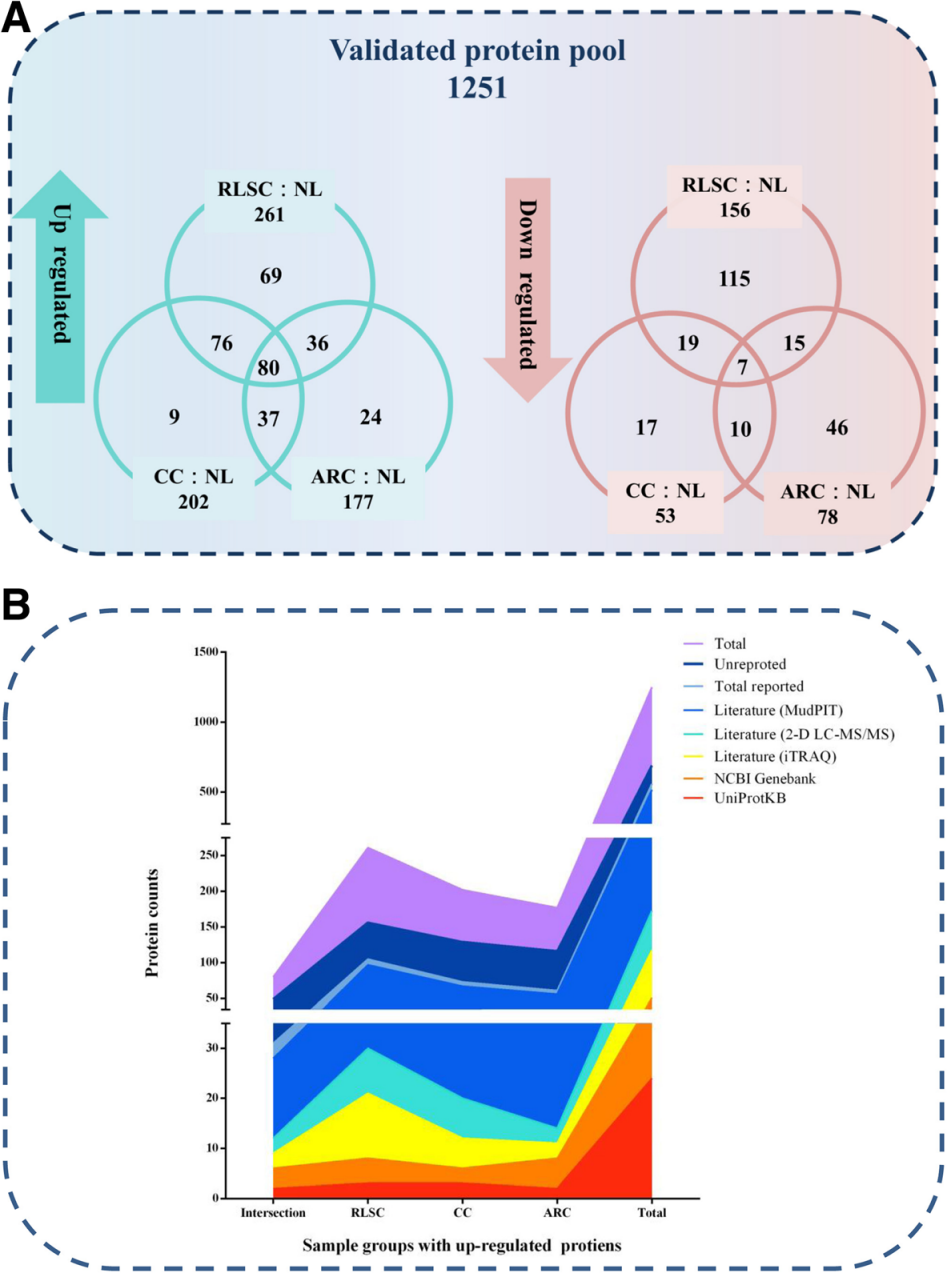
Differentially expressed proteins identified across the human RLSC, CC, ARC groups and an overview of previous reports. **a**. Venn diagram of the inter-relationship of the proteins identified in the human RLSC, CC and ARC groups. **b**. Overview of previous reports on proteins identified in human lenses

Compared to their abundance in the normal lens (control), the abundances of the 261, 202, and 177 proteins identified in the RLSC, CC and ARC groups, respectively, were significantly upregulated. (protein ratio ≥ 2, C.V ≤ 0.5), and 80 proteins were found in the overlapping areas. Most of the significantly up-regulated proteins in the CC (95.5%) and ARC (86.4%) groups overlapped, whereas the up-regulated proteins in the RLSC group was were more specific, with a lower overlapping percentage (73.6%). A preponderance of down-regulated proteins (156) was found, and the percentage of specific proteins (32.1%) in the RLSC groups was higher than those in the ARC (78, 59.0%) and CC (53, 32.1%) groups, as presented in the Venn diagram in Fig. 2a.

An overview of previous reports on the identified proteins and the up-regulated components in each group of human lenses is presented in Fig. 2b. A large majority of the proteins (55.2%, 691/1251) identified in the total protein pool have not previously been reported. Among the minor reported components, proteins identified using MudPIT constituted the largest proportion (92.3%, 517/560), followed by two-dimensional (2-D) LC-MS/MS identification [3] (30.9%, 173/560), and by iTRAQ [14] identification (21.1%, 118/560), National Center for Biotechnology Information (NCBI) GenBank records (9.3%, 51/560) and UniProtKB records (4.3%, 24/560). The RLSC (22.6%, 156/691), CC (18.7%, 129/691), and ARC (16.8%, 116/691) groups exhibited a degressive, unreported protein ratio.

### Bioinformatics functional annotation revealed common final pathways with diverse upstream regulation for cataractogenesis in different physiopathological states

The GO annotation analyses were performed for the 261, 202, and 177 significantly up-regulated proteins of the RLSC, CC and ARC groups, respectively, to elucidate their biological processes, molecular functions and cellular components.

#### GO biological process analysis

**Fig. 3:**
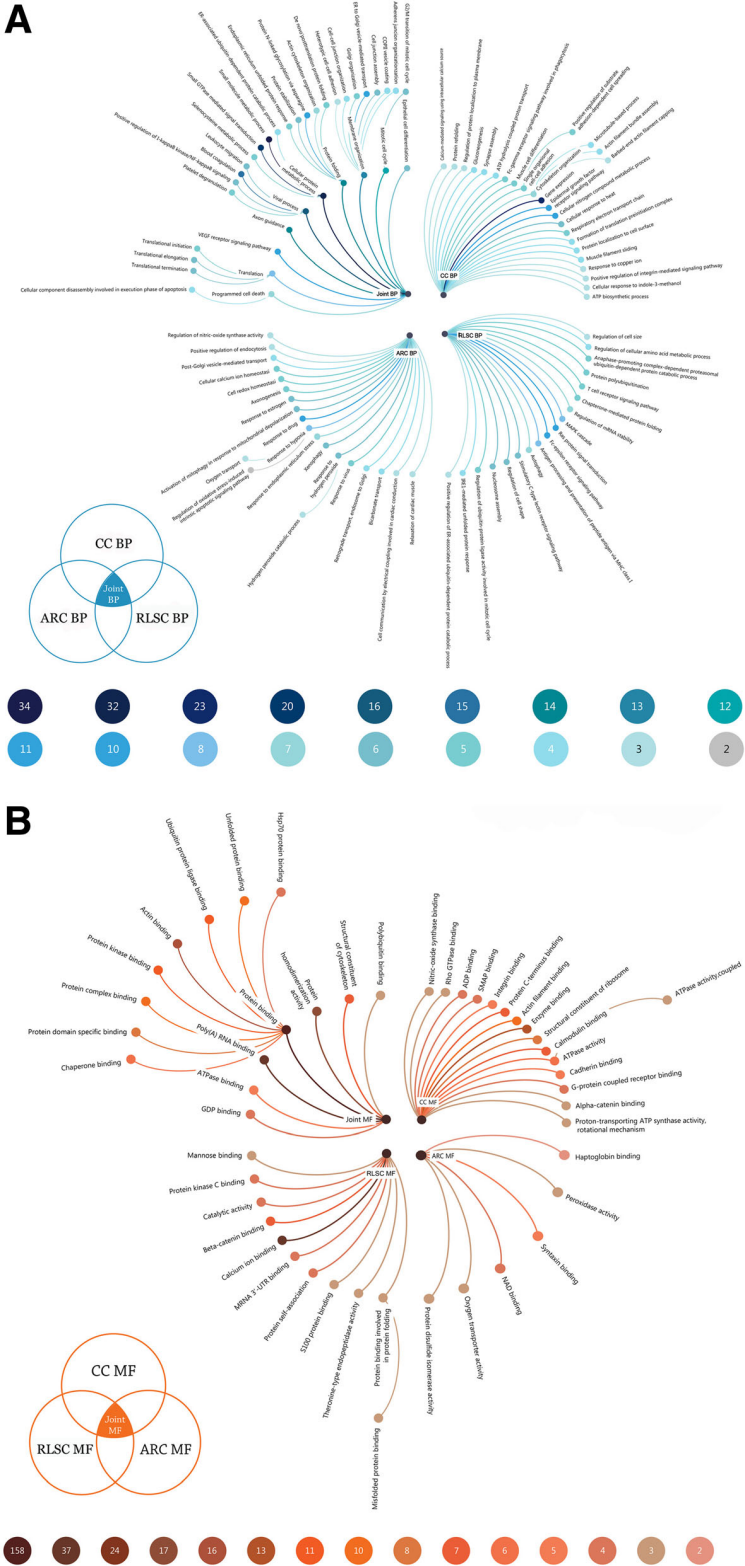
Radial tidy trees illustrating the significant GO items involved in the human RLSC, CC, and ARC groups and their joint components. The node texts represent the involved GO terms; the node colour gradients reflect the degree of enrichment of the corresponding GO terms. **a**. The significantly involved GO biological process (BP) items; **b**. The significantly involved GO molecular function (MF) items

*Cellular protein metabolic process*, *immune responsive process* and *protein folding-related biological processes* were the top enriched items shared by the RLSC (*n* = 32, 22, and 16, respectively; all *p* < 0.0001), CC (*n* = 37, 19, and 15, respectively; all *p* < 0.0001) and ARC groups (*n* = 37, 16, and 14, respectively; all *p* < 0.0001). In the RLSC group, the *Fc-epsilon receptor signaling pathway* was primarily involved (*n* = 10, *p* = 0.027), followed by *Ras protein signal transduction* (*n* = 10, *p* = 0.005) and *Antigen processing and presentation of peptide antigen* via *major histocompatibility complex (MHC) class I* (*n* = 8, *p* = 0.004). In the CC group, *gene expression* (*n* = 23, *p* = 0.005), the *epidermal growth factor receptor signaling pathway* (*n* = 11, *p* = 0.008), and the *cellular nitrogen compound metabolic process* (n = 10, *p* = 0.026) were the most predominantly enriched items. In contrast, in the ARC group, enrichment of the items *response to drug* (n = 11, *p* = 0.007)*, response to hypoxia* (n = 8, *p* = 0.006) and *response to endoplasmic reticulum stress* (*n* = 7, *p* = 0.0002) was observed (Fig. 3a).

#### GO molecular function analysis

The most significant enriched GO molecular function items shared by the RLSC (*n* = 169, 47, and 23, respectively; all *p* < 0.0001), CC (*n* = 161, 47, and 21, respectively; all p < 0.0001) and ARC (*n* = 158, 37, and 17, respectively; all *p* < 0.0001) groups were *protein binding, poly (A) RNA binding* and *protein homodimerization activity*, respectively. In the RLSC group, *calcium ion binding* was predominantly involved (*n* = 24, *p* < 0.0001), followed by *beta-catenin binding* (*n* = 7, *p* = 0.0007) and *MRNA 3’-UTR binding* (*n* = 4, *p* = 0.020). In CC group, *enzyme binding* (*n* = 13, *p* = 0.0009), the *structural constituents of ribosome* (*n* = 8, *p* = 0.017), and *calmodulin binding* (n = 7, *p* = 0.025) were the most predominantly enriched items. Finally, in the ARC group, *syntaxin binding* (*n* = 5, *p* = 0.015)*, nicotinamide adenine dinucleotide (NAD) binding* (n = 4, *p* = 0.011) and *oxygen transporter activity* (*n* = 3, *p* = 0.012) were observed (Fig. 3b).

#### GO cellular component analysis

The overwhelming majority of the enriched proteins were localized in the *extracellular exosome*, *cytoplasm*, *membrane* and the extended substructures in the RLSC (*n* = 132, 92, and 60, respectively; all *p* < 0.0001), CC (*n* = 121, 95, and 66, respectively; all *p* < 0.0001) and ARC (*n* = 119, 79, and 68, respectively; all *p* < 0.0001) groups. In particular, the cellular component item *nucleus was involved in* the *RLSC* (*n* = 79, *p* = 0.038) *and CC* (*n* = 74, *p* = 0.0374) groups but was not significantly involved in the ARC group.

#### KEGG analysis

KEGG pathway mapping revealed 10, 22 and 17 enriched pathways in the RLSC, CC and ARC groups, respectively. These groups shared the three most commonly affected pathways: *protein processing in the endoplasmic reticulum* (*n* = 16, 12, and 13; all *p* < 0.0001), *phagosome* (n = 11, 15, and 12; all *p* < 0.0001) and *regulation of the actin cytoskeleton* (all n = 16; *p* = 0.006, 0.009, and 0.006). In the *RLSC group, Huntington’s disease* (*n* = 8, *p* = 0.031) *and proteasome* (n = 8, *p* = 0.031) were specifically involved*.* In the *CC group, Focal adhesion (n = 9*, *p = 0.022), Leukocyte transendothelial migration (n = 8*, *p = 0.004),* and *ribosome (n = 8*, *p = 0.025)* were the most dominant enriched items. Finally, in the ARC groups, *Endocytosis (n = 9*, *p = 0.048), Salivary secretion (n = 6*, *p = 0.010) and Proximal tubule bicarbonate reclamation (n = 4*, *p = 0.005)* were specifically observed (Table 2).

**Table 2:**
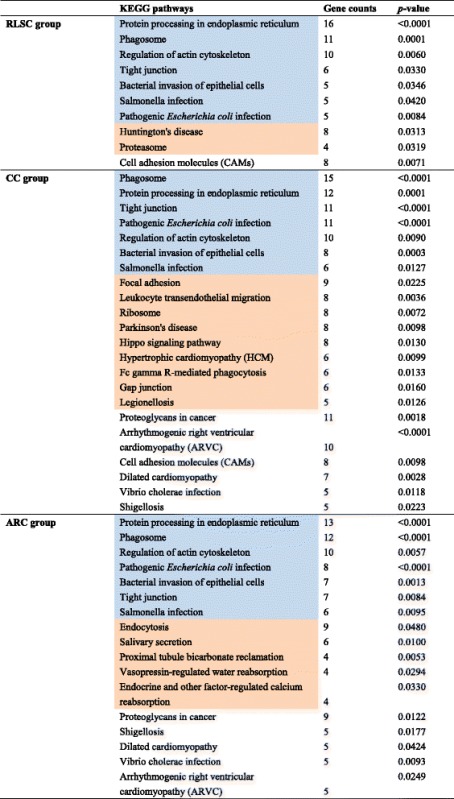
The KEGG pathways involved in RLSC, CC and ARC by the up-regulated proteins

Blue indicates the common pathways shared by the RLSC, CC and ARC groups and orange represents the unique pathways detected in the corresponding groups

### The unbalanced abundance of the representative cataractous proteins with relatively high sequence coverage between groups provides insights into the underlying molecular mechanisms in different physiopathological states

Comparing the relative abundances of the principal proteins between groups and the normal lens as a control, α-crystallin proteins showed no changes in different physiopathological conditions; β-, γ- and λ- crystallin protein families were broadly down-regulated, except for β-crystallin A2, which exhibited significant overexpression in the RLSC, CC and ARC groups.

Cytoskeletal proteins, including beaded filament structural protein (BFSP1), BFSP2, tubulin alpha 1a (TUBA1A), myosin heavy chain 9 (MYH9) and retinitis pigmentosa 2 (RP2), were predominantly up-regulated proteins in the ARC group but were less prominent in the RLSC and CC groups. Vimentin was prominently over-expressed in CC lenses. Thus, dysfunction, rather than inadequate amounts of these components, contributes to the intracellular cytoskeletal instability in the corresponding physiopathological states.

Abhydrolase domain containing 12 (ABHD12), which is a crucial metabolic enzyme, was most significantly over-expressed in the RLSC group, followed by the CC and ARC groups. Other metabolic enzymes, including lens protein with glutamine synthetase domain (LGSN), aldehyde dehydrogenase 1 family member A1 (ALDH1A1), apolipoprotein A1 (APOA1), sorbitol dehydrogenase (SORD), tyrosine 3-monooxygenase/tryptophan 5-monooxygenase activation protein epsilon (YWHAE+), apolipoprotein E (APOE), and lanosterol synthase (LSS), were down-regulated or slightly up-regulated in the RLSC, CC and ARC groups.

**Fig. 4:**
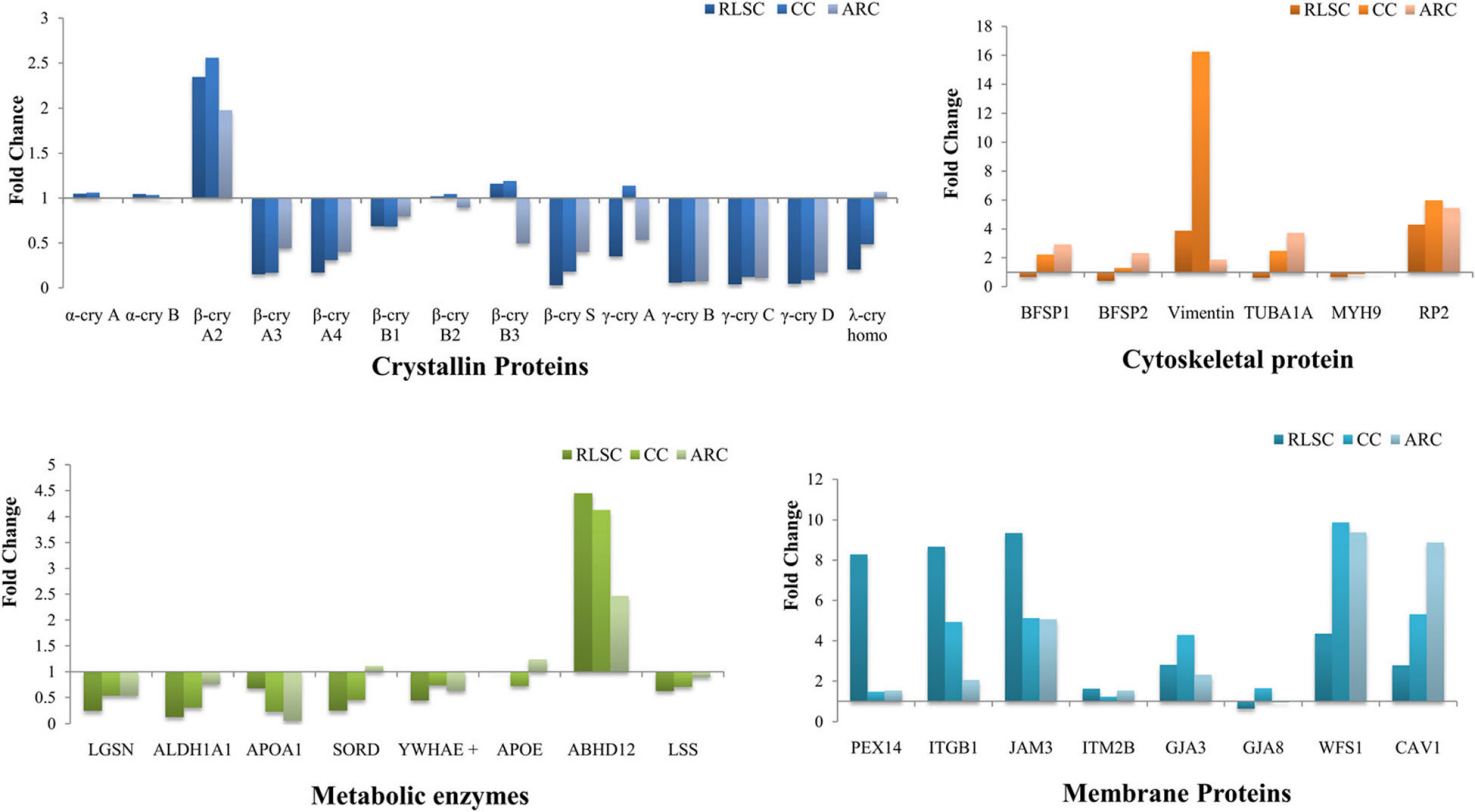
Relative abundance of the representative cataractous proteins with relatively high sequence coverage identified in the human RLSC, CC and ARC groups

Membrane proteins, including peroxisomal biogenesis factor 14 (PEX14), integrin subunit beta 1 (ITGB1), junctional adhesion molecule 3 (JAM3), integral membrane protein 2B (ITM2B), gap junction protein alpha (GJA3), GJA8, and wolframin ER transmembrane glycoprotein (WFS1), were generally up-regulated in the RLSC, CC and ARC groups. Further observation revealed that PEX14, ITGB1 and JAM3 were prominently up-regulated in the RLSC groups, indicating the potential involvement of intercellular adhesion in the regulation of lens regeneration. See Fig. 4 and Table 3 for details.

**Table 3:**
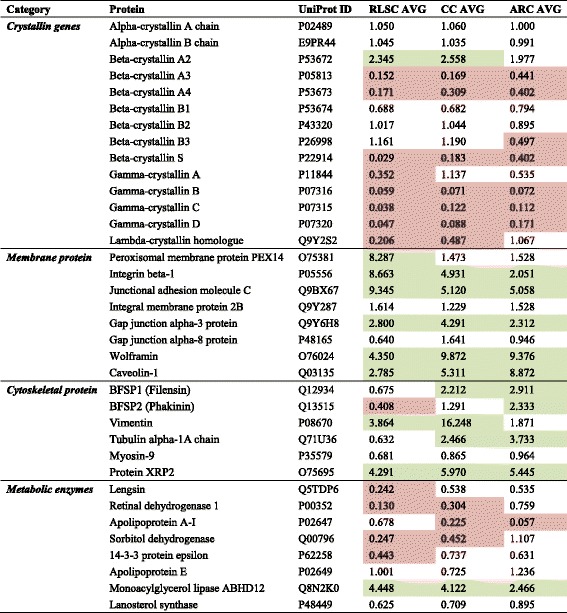
Relative abundances of the representative cataractous proteins identified in the RLSC, CC and ARC groups

Green represents the up-regulated proteins and red indicates the down-regulated proteins

### The protein-protein interaction network generated by the identified cataract-associated proteins in different physiopathological states

**Fig. 5:**
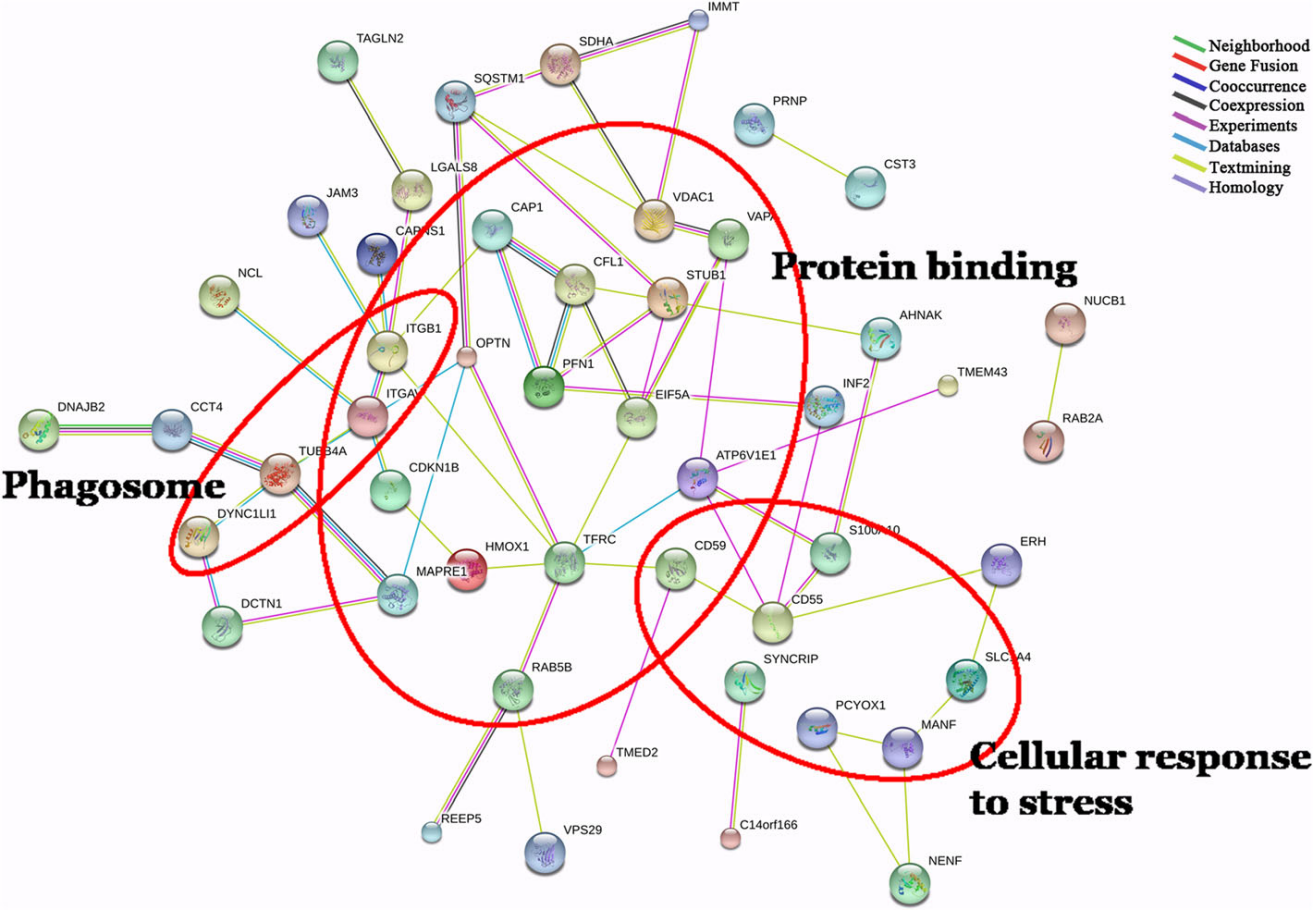
Protein-protein interaction network of the identified cataract-associated proteins in different physiopathological states

Using the STRING interaction database, the commonly up-regulated proteins shared by the RLSC, CC, and ARC groups were analyzed to illustrate the protein-protein interactions associated with cataractogenesis. Medium-confidence interactions (minimum required interaction score, 0.400) were set for the analysis. The common up-regulated proteins primarily comprised the protein networks, indicating the involvement of protein folding, response to stress and phagosome (Fig. 5).

### Proteogenomic characterization narrowed the list of candidate causal molecules

**Fig. 6:**
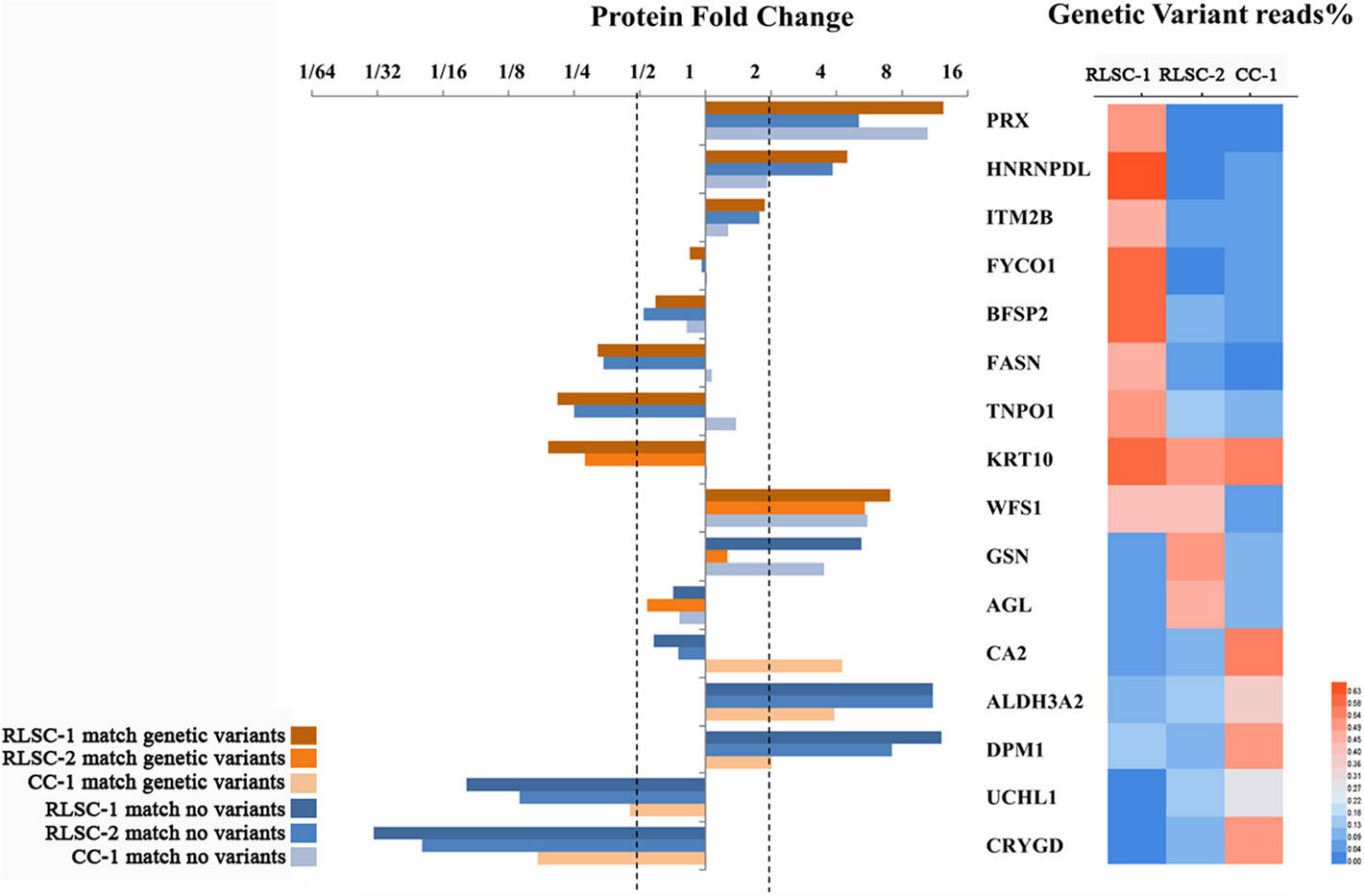
Proteogenomic characterization of subjects in the RLSC and CC groups performed to detect susceptibility genes/proteins

WES testing and analysis were performed for two individuals in the RLSC group and one individual in the CC group (Additional file 2: Table S2). After bioinformatics filtering from the raw sequencing data based on mutation types, low minor allele frequencies and functional predictions, 160 variants of the corresponding 125 genes were prioritized for further proteogenomic interpretation. Among the 125 variant genes, 16 corresponding proteins were simultaneously detected and validated using iTRAQ. In the RLSC group, 11 proteins were detected with the corresponding genetic variants, among which 3 proteins (peroxiredoxin [PRX], heterogeneous nuclear ribonucleoprotein D like [HNRNPDL] and WFS1) were significantly up-regulated (fold-change >2); another 3 proteins (fatty acid synthase [FASN], transportin 1 [TNPO1] and keratin 10[KRT10]) were significantly down-regulated (fold-change < 0.5). In the CC group, 6 proteins were detected with the corresponding genetic variants, among which 2 proteins (carbonic anhydrase 2 [CA2] and dolichyl-phosphate mannosyltransferase subunit 1 [DPM1]) were significantly up-regulated (fold-change >2); another 2 proteins (ubiquitin c-terminal hydrolase L1 [UCHL1] and crystallin gamma D [CRYGD]) were significantly down-regulated (fold-change < 0.5) (Table 4 and Fig. 6).

**Table 4:**
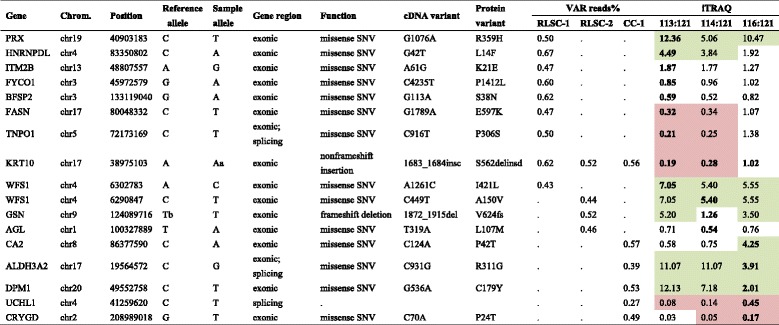
Proteogenomic analysis of subjects in the RLSC and CC groups performed to detect susceptibility genes/proteins

Green represents the up-regulated proteins and red indicates the down-regulated proteins

## Discussion

The present study focused on the quantitative comparison of the human lens proteomic data obtained from different physiopathological states (i.e., the RLSC, CC and ARC groups). The most complete human lens proteome, including 1251 proteins, was identified, and a large majority of the proteins were previously unreported. Bioinformatics functional annotation revealed the common involvement of cellular metabolic process, immune responses and protein folding disturbances, but diverse upstream regulation were observed for cataractogenesis in different physiopathological states. Combined with the detection of genetic variants using WES, the susceptibility genes/proteins were narrowed to a list of 16 molecules.

The differentially expressed proteins identified in the RLSC, CC and ARC groups overlapped in relatively large proportions, indicating the involvement of common underlying biological processes. Subsequently, the performance and interpretation of the GO analysis, KEGG pathways and STRING networks defined the common final pathways by extracting the common items among the individual groups. The common final biological processes for RLSC, congenital and senile cataracts were inferred to enable cellular metabolic processes, immune responses and protein folding disturbances. The underlying molecular mechanisms were multilevel, including transcriptional modification (*poly [A] RNA binding*) and post-translational protein interventions (*protein binding and protein homodimerization activity*). The main location of the common cataractogenesis function was predicted to be extranuclear zones, such as the extracellular exosome, cytoplasm, and membrane.

Interestingly, the RLSC, CC and ARC processes follow diverse paths but arrive at the same destination. The mysterious regulation of lens regeneration was associated with immune responses, and evidence of an enriched Fc-epsilon receptor signaling pathway, antigen processing and presentation of peptide antigens via MHC class I was found*.* Unlike the common final pathways, the specific process disposal component was indicated in the nucleus, with cellular signal transduction regulation by calcium ion and ubiquitin-proteasome pathways.

The biological processes of the CC group were associated with gene expression and vascular endothelial growth factor (VEGF) signaling pathways, confirming the involvement of genetic predisposition and development dysregulation [15]. Age-related cataractogenesis featured responses to external stimuli, including hypoxia, drugs, estrogen, and viruses. In contrast to RLSC and CC, ARC involved specific processes localized to the extracellular area with rare interruption of the genetic materials. As previously suggested, this finding could indicate that fetal lenses, as in the RLSC and CC groups, contain more cells with relatively complete cellular structures, including the nucleus and organelles, and therefore occupy the biological process and molecular pathways involving the DNA and RNA within the nucleus [3].

The expression of most crystallin proteins, including α, β, and γ crystallin proteins was down-regulated in regenerative lens, congenital and senile cataracts compared with that in normal lenses, consistent with the findings of Su et al. [16]**.** The involved mechanism was suggested to be the evident aggregation [17, 18] and degradation [19] of crystallins during cataract formation. An exception was β-crystallin A_2_, which exhibited significant up-regulation in the RLSC, CC and ARC groups with the normal lens as the control. β-crystallin A_2_ was not included in the identification of human crystallins in previous proteomics reports. The dynamic expression of the crystallin components and its implications remains elusive.

CC is a representative disease with substantial genetic heterogeneity and manifested complexity [20, 21]. Genomic sequencing failed to detect the causal genetic variants in sporadic patients, who constitute more than two thirds of the total number. After linking the genome to the proteome in the present study, hundreds of candidate genetic variants and thousands of differentially expressed proteins were eliminated to create a list of 16 genes and their corresponding proteins. Thus, this methodology is useful for simplifying the complexity of isolated genomic or proteomics data and could support the development of precision medicine, particularly for diseases with high genetic and clinical heterogeneity [22].

### Study strengths and limitations

The present study should be interpreted within the context of its strengths and limitations. The unique RLSC samples, the most complete human lens proteome identification to date and the comprehensive multilayer bioinformatics interpretations demonstrate not only the strength but also some of the limitations inherent to the present study. The regenerative lenses were obtained from our novel minimally invasive surgical procedures, and transparency was maintained in most subjects. Thus, the number of samples in the RLSC group was too small, and experimental replicates or validations were not feasible. Additionally, all samples were collected during minimally invasive surgical procedures. However, it was not realistic to obtain the entire lens contents or the specific lens zone subjected to extracapsular cataract extraction (ECCE).

Nevertheless, because a quality control strategy was used for proteomics identification and because each surgery was performed by an experienced surgeon, the findings contribute to elucidating the mechanism underlying cataractogenesis with respect to diverse crystallin lens dysregulated models. Subsequent studies involving larger sample sizes and experimental validation will be conducted to confirm and further explore the target molecular pathways. Moreover, the proteogenomic analysis conducted here has great favorable translational potential for detecting candidate susceptibility genes/proteins and, thus, facilitating the development of precision medicine, particularly for diseases with high genetic and clinical heterogeneity.

## Conclusions

These findings revealed that regenerative lens proteome composition was distinct from that of others. Cataractogenesis shares common final pathways with diverse upstream regulation of cataractogenesis in different physiopathological states. The proteogenomic analysis narrowed the list of candidate susceptibility genes/proteins, showing translational potential for detecting candidate causal molecules in precision medicine.

## Additional files

Additional file 1: Table S1. Identification and quantification of total proteins across the RLSC, CC and ARC groups. (XLSX 217 kb)

Additional file 2: Table S2. Identification of genetic variants and filtering analysis for the RLSC and CC groups. (XLSX 22 kb)

## Abbreviations

ARC: Age-related cataract
CC: Congenital cataract
ECCE: Extracapsular cataract extraction
GO: Gene Ontology
iTRAQ: Isobaric tagging for relative and absolute protein quantification
KEGG: Kyoto Encyclopedia of Genes and Genomes
LECs: Lens epithelial stem cells
RLSC: Regenerative lenses with secondary cataract
STRING: Search Tool for the Retrieval of Interacting Genes/Proteins
WES: Whole Exome Sequencing
